# O desenvolvimento da identidade de gênero e a construção de si de mulheres transgênero durante a juventude

**DOI:** 10.1590/0102-311XPT082725

**Published:** 2026-04-27

**Authors:** Náila Neves de Jesus, Aline Silva de Assis Santos, Mariana Martins Gonzaga do Nascimento, Rita Maria Radl-Philipp

**Affiliations:** 1 Universidade Federal de Minas Gerais, Belo Horizonte, Brasil.; 2 Universidade Estadual do Sudoeste da Bahia, Vitória da Conquista, Brasil.

**Keywords:** Adolescente, Pessoas Transgênero, Identidade de Gênero, Adolescent, Transgender Persons, Gender Identity, Adolescente, Personas Transgénero, Identidad de Género

## Abstract

O objetivo foi analisar como mulheres transexuais e travestis, na Bahia (Brasil), narram a constituição de suas identificações de gênero na juventude e a construção de corporalidades, articulando memória coletiva e normas de gênero. Estudo qualitativo em duas cidades (interior e capital), com 12 participantes nomeadas por pseudônimos. Realizaram-se entrevistas semiestruturadas (2020-2021), diário de campo e análise de conteúdo temática (Bardin), com gerenciamento no Atlas.ti, dupla codificação, triangulação e revisão por pares. Os relatos evidenciaram o papel da família, da escola, da religião e do território como quadros sociais de memória que estruturam lembranças; mostraram também como a hormonização precoce, o uso de tecnologias corporais e a experiência de patologização conformaram negociações de reconhecimento e acesso ao cuidado. Além disso, as narrativas destacaram a produção de nomes e posicionamentos identitários como atos performativos situados. A memória opera como prática social regulatória e como potência performativa de resistência, o que implica políticas de saúde que superem a patologização e reconheçam especificidades de travestis e mulheres transexuais.

## Abstract (en)

The aim was to analyze how transsexual women and *travestis* in the state of Bahia, Brazil, narrate the constitution of their gender identification in youth and the construction of self, linking collective memory and gender norms. A qualitative study was conducted in two cities (capital and instate municipality) with 12 participants identified by pseudonyms. Data collection involved interviews (2020-2021), a field log, and thematic content analysis (Bardin), with management in Atlas.ti, double coding, triangulation, and peer review. The reports revealed the role of family, school, religion, and area of residence as social frameworks that structure memories, and showed how early hormone treatment, the use of body technologies, and the experience of pathologization shaped negotiations for recognition and access to care. Moreover, the narratives highlighted the production of names and identity positionings as situated performative acts. Memory operates as a regulating social practice and a performative power of resistance, which implies the need for health policies that overcome pathologization and recognize the specificities of *travestis* and transsexual women.

## Abstract (es)

El objetivo del estudio fue analizar cómo las mujeres transexuales y travestis del estado de Bahía (Brasil) narran la constitución de sus identificaciones de género en la juventud y la construcción de corporalidades, articulando la memoria colectiva y las normas de género. Se trata de un estudio cualitativo realizado en dos ciudades (en el interior y en la capital), con 12 participantes identificadas por seudónimos. Se realizaron entrevistas semiestructuradas (2020-2021), un diario de campo y un análisis de contenido temático (Bardin), con gestión en Atlas.ti, doble codificación, triangulación y revisión por pares. Los relatos pusieron de manifiesto el papel de la familia, la escuela, la religión y el territorio como marcos sociales de memoria que estructuran los recuerdos; también mostraron cómo la hormonización precoz, el uso de tecnologías corporales y la experiencia de patologización conformaron las negociaciones de reconocimiento y acceso a la atención. Además, las narrativas destacaron la producción de nombres y posicionamientos identitarios como actos performativos situados. La memoria opera como práctica social reguladora y como potencia performativa de resistencia, lo que implica políticas de salud que superen la patologización y reconozcan las especificidades de las travestis y las mujeres transexuales.

## Introdução

Os processos de construção de identificações de gênero de pessoas trans têm sido atravessados por normativas sociais que delimitam corpos, comportamentos e trajetórias possíveis ^1^. Em nossa sociedade, marcada por estruturas patriarcais, cis e heteronormativas, tais identificações frequentemente desafiam expectativas culturais e encontram resistências institucionalizadas em diferentes esferas da vida social ^2^. Empregamos cis-heteronormatividade para designar o conjunto de normas, expectativas e dispositivos que naturalizam a cisgeneridade e a heterossexualidade como padrão legítimo de vida, regulando inteligibilidade, pertencimento e acesso a direitos.

A identidade travesti e a identidade de mulher transexual, embora compartilhem experiências de marginalização, constituem categorias políticas, sociais e subjetivas distintas. Ambas enfrentam violências simbólicas e materiais, mas suas trajetórias são marcadas por sentidos e performatividades singulares. Consideramos essencial explicitar essas distinções desde o início, pois elas são frequentemente invisibilizadas no discurso biomédico e nas práticas dos serviços de saúde. Por diversas vezes, a categoria travesti foi associada a abjeção, prostituição e criminalidade, enquanto a mulher transexual é uma identidade que funciona como uma espécie de higienização social e purificadora, fortemente relacionada ao discurso medicalizante e patologizante ^3^ ^,^ ^4^.

Destarte, a terminologia “transgênero” ou “transgeneridade” é um guarda-chuva que abarca diferentes expressões identitárias ^5^. Como argumenta Hall ^5^, identidades não são essências fixas, mas processos relacionais, sempre em disputa, negociados no campo cultural. Dessa forma, “transgeneridade” aqui não funciona como categoria universalizante, mas como operador político que permite visibilizar múltiplas experiências identitárias. Ainda, sob a percepção de Hall acerca da construção de identidades, temos processos de identificação - contingentes, mutáveis e situados - sustentados por repertórios discursivos e marcos de memória que variam conforme pertenças e conflitos ^6^.

Adicionalmente, toda identidade é relacional, construída a partir da diferença - isto é, não existe fora do contraste com aquilo que a sociedade toma como norma ^6^. A partir do momento em que pessoas transgênero rompem com padrões cis-heteronormativos, entretanto, há um processo de invisibilização e discriminação social ^7^.

O estigma não deriva de uma “aversão ao diferente”, mas de processos sociais que definem diferenças como marcas desqualificantes, rebaixando o *status* e reconfigurando interações ^8^. Avtar Brah ^8^ ressalta que a diferença não pode ser reduzida à diversidade cultural, mas deve ser entendida como uma relação de poder. Assim, as identidades trans tensionam as fronteiras sociais do sexo/gênero ao evidenciar como os significados culturais sobre a sexualidade e o corpo são produzidos por sistemas dominantes de representação.

Isto reflete também na dificuldade que essas pessoas têm em acessar direitos básicos e no fato de sofrerem diversos tipos de violência, o que as marginaliza socialmente, e, frequentemente, ceifa-lhes a vida ^7^. Como representação dessa realidade, observa-se que o Brasil lidera o ranking mundial de assassinatos de pessoas transgênero pelo 15º ano consecutivo, registrando 122 assassinatos em 2024, majoritariamente associados a atos de crueldade ^9^.

A juventude transgênero é o estrato mais atingido por violências, sendo que 48% dos assassinatos ocorrem na faixa etária de 18-29 anos ^8^. Portanto, mesmo com importantes avanços nos direitos LGBTQIAPN+, jovens trans e travestis são impossibilitados de se desenvolver e existir com segurança e qualidade de vida ^10^. Como enfatiza Crenshaw ^9^, a interseccionalidade revela que a vulnerabilidade dessas juventudes não pode ser explicada por um único eixo (gênero ou sexualidade), mas pelo entrecruzamento de opressões de classe, raça e gênero que se potencializam mutuamente.

Na mesma senda, é importante também considerar a constituição das identidades de gênero como um resultado da construção social mediante apreensão de modelos internalizados durante a vida pessoal, que inclui o tensionamento social e ciclo de violência contínuo. Halbwachs ^11^ contribui aqui ao indicar que a memória nunca é individual, mas sempre social, constituída nos quadros coletivos em que os sujeitos estão inseridos. Assim, o uso do termo “indivíduo” neste estudo não remete a uma essência isolada, mas a um sujeito situado em redes de memória e pertencimento social.

Para este autor, lembrar é sempre lembrar-com: as narrativas juvenis se apoiam em quadros sociais (família, escola, religião, cidade) que atualizam o passado segundo as necessidades do presente do grupo, orientando o que se torna dizível e o que permanece silenciado. Esses quadros não são estáticos: atualizam o passado à luz das necessidades do presente do grupo, fornecendo esquemas de interpretação que tornam certas lembranças comunicáveis e outras impronunciáveis ^12^.

Não obstante, estes mesmos componentes são responsáveis pela construção das identidades sociais. Connerton ^13^ complementa ao afirmar que o corpo é também lugar de memória, o que permite compreender como as transformações corporais narradas por travestis e mulheres trans - sejam elas decorrentes da hormonização, da violência ou da performatividade de gênero - constituem práticas de lembrança e inscrição de suas trajetórias.

Essa chave halbwachsiana conversa diretamente com a crítica de Butler ^14^ ao gênero como dispositivo regulatório: não há identidade fora da norma; há sujeitos constituídos por repetições performativas que estabilizam o reconhecível. Se a norma de gênero regula as fronteiras do quem pode ser e como deve aparecer, os marcos sociais da memória regulam o que se pode lembrar e de que modo narrar. Ao aproximar estes dois autores tratamos as recordações não como registros neutros, mas como efeitos de enquadramento - normativos e disputados - que incidem sobre a possibilidade mesma de contar uma juventude trans ^12^ ^,^ ^14^.

Dessa forma, este estudo buscou analisar aspectos relacionados ao processo de reconhecimento da identidade de gênero e à construção do corpo compatível com a identidade entre mulheres trans e travestis a partir de sua memória e percepção sobre o período juvenil.

## Métodos

### Desenho do estudo

Realizamos um estudo qualitativo orientado pelo referencial de memória coletiva e marcos sociais da memória (Halbwachs), que estruturou tanto o roteiro de entrevistas quanto a interpretação dos dados. Esse enquadramento permitiu reconstruir, a partir do presente, as lembranças da juventude e seus marcos (família, religião, espaço, tradições, linguagem, tempo) implicados nas identificações de gênero. Foi utilizado o apoio teórico-metodológico dos preceitos sobre memória coletiva e, principalmente, sobre os marcos sociais da memória de Maurice Halbwachs ^11^ ^,^ ^12^. Portanto, partiu-se da coleta de relatos e narrativas reconstruídas e resgatadas pelas participantes, que tecem o fio da memória a partir de marcos sociais que operam no processo de recordação.

### Contexto social do estudo e participantes

O estudo ocorreu em duas cidades do Estado da Bahia de perfis distintos (interior e capital), favorecendo contrastes territoriais relevantes. Incluímos mulheres trans e travestis que relataram vivências da juventude relacionadas a corpo, acesso à saúde e sociabilidades. Participaram 12 pessoas, recrutadas por contatos institucionais e redes das próprias participantes. Os critérios de inclusão contemplaram autoidentificação como mulher trans ou travesti e maioridade; a exclusão restringiu-se a recusas ou impossibilidade de consentimento.

As mulheres entrevistadas receberam nomes fictícios: Esmeralda, Ametista, Jade, Rubi, Safira, Turmalina, Topázio, Turquesa, Água Marinha, Diamante e Cristal. Os nomes de pedras preciosas representam a beleza única e preciosidade de cada uma das entrevistadas que, assim como pedras preciosas, são difíceis de encontrar. O contato e a aproximação com as participantes constituíram um caminho permeado de percalços e dificuldades. Entendemos que não é fácil para elas confiar, se abrir e compartilhar suas memórias juvenis em vista de suas marcas do passado. Mas, ao encontrarmos estas pedras, o valor e a beleza de seu brilho transparecem e reluzem. Suas características sociodemográficas e das entrevistas estão descritas no Quadro 1.

**Quadro 1:** Características relatadas pelas participantes e detalhes da entrevista.

PARTICIPANTES	IDENTIDADE DE GÊNERO	IDADE	ESTADO CIVIL	NÍVEL DE EDUCAÇÃO	PROFISSÃO/OCUPAÇÃO	LOCAL DE RESIDÊNCIA	DURAÇÃO DA ENTREVISTA
Esmeralda	Mulher trans	46	Solteira	Não informado	Pensionista	Vitória da Conquista	67min53s
Ametista	Mulher trans	51	Solteira	Superior completo	Professora	Vitória da Conquista	45min12s
Jade	Mulher trans	46	Solteira	Não informado	Garçonete	Vitória da Conquista	29min39s
Rubi	Travesti	30	Solteira	Superior completo	Escritora/Poetisa/Garota de programa	Vitória da Conquista	15min25s
Safira	Travesti	35	Solteira	Ensino Médio	*Linkadora*	Salvador	45m23s
Turmalina	Mulher trans	32	Casada	Não informado	Cozinheira/*Bartender*	Salvador	45m23s
Ágata	Travesti	34	Solteira	Não informado	Manicure	Salvador	30m15s
Topázio	Travesti	26	Solteira	Superior incompleto	Cuidadora de idosos	Salvador	30m15s
Água Marinha	Travesti	37	Casada	Analfabeta	Garota de programa	Salvador	31m54s
Turquesa	Mulher trans	21	Solteira	Superior incompleto	Estudante universitária	Salvador	31m54s
Diamante	Travesti	37	Solteira	Não informado	Artista/Performista	Salvador	34m45s
Cristal	Mulher trans	47	Solteira	Superior completo	Professora	Salvador	34m45s

### Coleta e organização dos dados

Na coleta de dados, adotou-se a entrevista semiestruturada em profundidade de modo presencial baseada no modelo do *Estudo Pop Trans* ^15^. Durante as entrevistas, foi explorado o processo de construção da identidade de gênero e suas interlocuções com as experiências nos espaços sociais, e com os marcos sociais da memória. Foram abarcadas experiências recordatórias na casa familiar e no contexto da família. Também foram realizadas questões em profundidade sobre a evolução histórica dos sentimentos, percepções e imagens em relação ao corpo e à identidade de gênero.

Para oferecer conforto, ficou a critério das participantes a escolha do local das entrevistas que ocorreram entre janeiro e fevereiro de 2020 e, posteriormente, em maio de 2021, em vista do contexto da pandemia provocada pelo COVID-19. Todas as entrevistas foram gravadas e depois transcritas, considerando as anotações do diário de campo da pesquisadora.

### Análise dos dados

Adotamos análise de conteúdo temática (Bardin) em três etapas: pré-análise, exploração e tratamento/interpretação. Utilizamos o Atlas.ti (http://atlasti.com/) para organizar o *corpus*, codificar unidades de significado, agrupar códigos e inspecionar coocorrências entre eixos (p.ex.: corpo/saúde; família/religião; escola/violência). As categorias emergentes foram derivadas dos núcleos de sentido identificados nas falas. A saturação teórica foi reconhecida quando novas entrevistas deixaram de adicionar códigos/temas relevantes às categorias em desenvolvimento. Garantimos credibilidade e confiabilidade por meio de: (i) leitura dupla/revisão por pares dos códigos e categorias; (ii) triangulação com diário de campo; (iii) trilha de auditoria (memórias analíticas no Atlas.ti). As divergências foram resolvidas por consenso.

### Aspectos éticos

O estudo foi aprovado pelo Comitê de Ética e Pesquisa da Universidade Estadual do Sudoeste da Bahia (parecer nº 3.821.210; CAAE 27988620.9.0000.0055). Antes da entrevista, as participantes receberam orientações e assinaram o Termo de Consentimento Livre Esclarecido. As identidades das participantes foram mantidas em sigilo.

## Resultados e discussão

### Construção inicial das identificações

Observou-se que a construção da identificação das participantes da pesquisa começa na infância, quando a pessoa percebe que não se enquadra nos papéis de gênero esperados para o sexo que lhe foi atribuído ao nascer. O processo de entendimento da identificação de gênero perpassou por várias fases entre as entrevistadas, com pontos de destaque que demarcaram sua construção da travestilidade: atração homoerótica, feminilidade da personalidade, identificação com brincadeiras, vestimentas e adornos “de menina”, entre outros.

Obviamente, os “signos de feminilidade” apreendidos pelas indivíduas durante suas vidas configuram a relação com os diversos marcos sociais da memória, na medida que vão para escolas, locais de trabalho, participam de grupos religiosos, desfrutam de espaços de lazer e da convivência no núcleo familiar. São nesses momentos de interação com estes elementos que a identificação de gênero também é forjada, conforme observamos nas suas narrativas:

“*Minha infância foi muito ‘não normal’, porque eu sentia que eu tinha alguma coisa de estranho por não gostar de brinquedos de meninos, de sempre ter atrações por meninos, não por meninas. E aí eu fui... tinha aquela... aquela dificuldade. Porque, naquela época, não se falava em transgeneridade, né? Se falava muito de travesti e gay, e, com meus 14 anos, eu não sabia o que eu era. Eu não... não entendia o que eu era*” (Safira, 35 anos).

“*Eu me descobri aos cinco anos de idade. Sempre, desde pequeninha, eu sempre dizia... eu dizia assim: ‘poxa! Não. Quando eu crescer, eu vou ser uma mulher.’ E sempre brincava, vestia roupa de mulheres*” (Cristal, 47 anos).

“*Eu me identifiquei com 12 pra 13 anos, que eu já tinha convicção da pessoa que eu era; da mulher que eu era*” (Ágata, 34 anos).

Destaca-se a recordação de Safira ao mencionar que não viveu uma infância “*normal*” por se sentir deslocada ou diferente das demais crianças. Essa recordação conecta-se ao que Santos ^16^ descreve como “*infância roubada*”, na qual comportamentos considerados desalinhados ao gênero esperado são reprimidos por pais e familiares, deixando marcas que ressurgem na vida adulta.

Compreende-se que há também, durante essa descoberta da (trans)identidade, um conflito com os marcos sociais da memória, principalmente a família e religião, nos quais há a transmissão de valores que condenam a transgeneridade e a homossexualidade. Imersas nesses valores, as entrevistadas relatam sofrer, inicialmente, com a culpa de que sua identidade e a orientação sexual são um pecado; um erro.

“*Quando eu fui crescendo, aos nove anos, eu fui para uma escola maior. E aí, começaram a me chamar de gay. Até então, eu não sabia o que era gay. Mas, está me chamando de gay? Tá bom. Então foi. E aí, um dia, o meu próprio irmão chegou para mim e falou: ‘você sabe o que é gay? É homem que gosta de homem’. Nessas alturas, eu já estava apaixonada por um coleguinha da escola e eu falei: meu Deus, eu sou gay e eu gosto de um menino. E aí veio o conflito da igreja, né?*” (Ametista, 51 anos, professora).

A memória aqui não é mero depósito de fatos, mas prática normativa, que reinscreve o gênero pela punição e pela vigilância. Halbwachs ^12^ nos lembra que tais recordações só existem porque se apoiam em quadros sociais - família, religião, escola - que estruturam o que pode ser lembrado e o que deve ser silenciado.

Neste sentido, a teoria do interacionismo simbólico proposta por George H. Mead indica que o ser humano só existe como sujeito a partir das suas relações sociais e em sua faceta de identidade pessoal, ou seja: “*não podemos ser nós mesmos, a menos que sejamos também membros em que haja uma comunidade de atitudes que controlam as atitudes de outros*” ^17^ (p. 192). Isto é, a constituição da identidade de gênero de uma pessoa perpassa por essas ações intersubjetivas, seus aspectos culturais e sociais. Assim, a interação com o outro e com as normativas sociais comum àquele grupo, em consonância com as estruturas sociais (linguagem, tradições, convenções sociais), é o que leva a pessoa a desenvolver uma consciência de si delineada por uma percepção que tem como referência o conjunto do seu entorno.

Por sua vez, a memória coletiva se ancora nas representações coletivas, que inclui os “*signos de feminilidade*” e de “*pecado*” citados pelas entrevistadas, e compõe as imagens do passado que a pessoa recorda a partir dos quadros sociais da memória ^18^. Nesse sentido, estes quadros sociais da memória atuam como pontos de referência para a recuperação do passado, fazendo com que nossas memórias individuais apresentem componentes do coletivo. O indivíduo precisa reconstrui-la a partir de instrumentos presentes no meio social, oriundos das convenções coletivas, na medida em que “*cada memória individual é um ponto de vista sobre a memória coletiva*” ^11^ (p. 69). Assim, todo o processo de construção das memórias está sujeito a uma representação coletiva do tempo e do espaço, que é culturalmente variável e historicamente construída ^19^.

Desta forma, é possível perceber que ambos os autores partem de um conceito de memória coletiva baseado em uma visão intersubjetiva, comunicativa e interacionista da memória. Em se tratando da identidade, a memória também é um elemento fulcral para a sua constituição, sendo um “*fato ligado à identidade da pessoa e como tal, funciona aquém da dinâmica das relações sociais dos sujeitos, segue uma dinâmica interativa comunicativa*” ^18^ (p. 39).

Este processo de amadurecimento, vem acompanhado de vivências de violência e discriminação que se iniciam nesse ambiente familiar a partir do momento em que iniciam a se comportar fora dos padrões ditos “*normais*” ou que “*saem do armário*” como gays afeminados, ou já como mulheres transgênero. Butler ^14^ mostra que as normas de gênero não apenas restringem, mas produzem sujeitos inteligíveis. Nesse caso, a inteligibilidade se dá pela exclusão: só é reconhecível quem corresponde ao padrão cis-heteronormativo. Hall ^5^ complementa ao afirmar que a identidade é processo narrativo em disputa. As memórias familiares das participantes revelam justamente esse embate entre discursos hegemônicos e resistências subjetivas.

“*A minha infância, né, fui uma criança muito afeminada* [risos]*. A minha família ficava falando pra minha mãe: ‘esfrega lá na cara, pra ver se vira homem, pega jeito, fala grosso, anda que nem homem’. E eu sempre me tratei no feminino* (...)*. E mainha ficava revoltada, né? Porque ela dizia que era a educação que ela deu: ‘meu filho é educado’. Não! Não é nada não*” (Turmalina, 32 anos).

“*Desde os nove anos, eu me visto de mulher e tudo, minha família ficou um pouquinho... não gostou, mas depois começou a se adaptar, normal, né, como ela disse que na rua a gente sofre verbalmente, né, xingada, é isso, mas a gente é só não ligar e fingir que nada aconteceu e seguir em frente*” (Topázio, 26 anos).

A linguagem - como um marco elementar da memória - também é um elemento que demarca o gênero e sempre privilegia a figura do homem em um lugar de autoridade. Além da linguagem, o simbolismo de gênero também opera nas roupas, maquiagem, gestos, fotografias, filmes, entre outros ^20^. Assim, vamos criando, a partir do que nos rodeia, os sentidos de feminilidade e masculinidade, e do que deve ser rechaçado e violentado se houver rompimento do acordo social comportamental normativo.

Dessa forma, estes papéis estereotipados reproduzidos através das narrativas das participantes e recordações de interações com a família se baseiam na identidade de gênero construída socialmente a partir dos papéis atribuídos à identidade feminina: reprodução, cuidado, docilidade, preocupação com a beleza e vivência no espaço doméstico. Estes significados estão presentes nas memórias do grupo mulheres de acordo com as reflexões de Elizabeth Jelín ^19^, sendo comum que mulheres construam recordações a partir de marcos familiares, visto que seu tempo subjetivo está organizado e ligado aos vínculos afetivos constituídos por meio da concepção do papel tradicional e estereotipado da mulher baseado no amor abnegado, no cuidado em atender ao próximo, sobretudo no âmbito da família.

O espaço também exerce influência na “*saída do armário*” das travestis. Através do relato de Rubi, percebemos que o contexto interiorano a reprimiu de assumir a identidade gay, e, em seguida, travesti quando era mais jovem:

“*Sempre omiti minha identidade por ser eu mesmo. Por ser cidade... por ser uma cidade de interior, eu sempre percebi que houve uma rejeição, sabe? Por parte, né, das pessoas em entender esses tipos de... de... comportamento identitários, né?* (...) *Eu demorei um tempo pra me assumir, só na universidade que eu tive, sei lá, uma... conseguir ter independência financeira, e tudo mais*” (Rubi, 30 anos).

De acordo com Arcos ^21^, o espaço opera como um ator importante na organização hierárquica das relações de gênero, classificando de acordo com a norma quem ocupa o seu centro e quem pertence à margem. O espaço é uma dimensão importante que exerce influência no modo como as pessoas são percebidas nas instituições sociais. Nesse ínterim, o espaço pode atuar como facilitador ou dificultador do trânsito dessas mulheres. A maioria das travestis que viviam no interior, por exemplo, tinha como objetivos futuros querer casar-se, cuidar dos familiares idosos e ter filhos. Enquanto as entrevistadas da capital Salvador já tinham perspectivas mais emancipatórias, como formar-se, ser independentes e lutar pelos direitos da comunidade trans em ativismo e movimentos sociais.

Esses marcos sociais da memória - sobretudo família e religião - produzem regimes de inteligibilidade que tornam certas lembranças juvenis comunicáveis apenas mediante traduções normativas (diagnóstico, “nome correto”), explicando itinerários de autocensura e a busca por mediações biomédicas de reconhecimento.

Nas falas das entrevistadas, percebemos também que, por diversas vezes, o reconhecimento como transexual ou travesti está relacionado, em suas memórias, ao diagnóstico psicológico/psiquiátrico:

“*Antes disso, eu tive que fazer um tratamento no Afrânio Peixoto para provar que eu tinha... é... um transtorno mental.* (…) [Após o diagnóstico de disforia de gênero] *Gente, agora eu sei o que eu sou, eu sou um transgênero, tava lá escrito. E eu cheguei na escola, mostrando às meninas que eu sou louca! Posso fazer o que eu quiser...*” (Ametista, 51 anos).

É curiosa a escolha das palavras que Ametista ao lembrar de um episódio com suas amigas, após o recebimento do diagnóstico de disforia de gênero que a categorizava como mulher trans. A suposição da patologização tão fortemente representada pelo diagnóstico a faz se considerar uma “*louca*” (também fazendo uma alusão ao hospital Afrânio Peixoto, que foi uma unidade de cuidado psiquiátrico na localidade da entrevista). A violência, a loucura e o diagnóstico atravessam a experiência trans com a presença obrigatória da nomeação da transexualidade como um “*desvio*”, pois segundo Butler ^22^ (p. 116), “*o diagnóstico assume que as normas de gênero são relativamente fixas e que o problema é assegurar que você encontre o que é certo, aquela norma que não fará você se sentir inadequada onde você está, confortável no gênero que é o seu*”.

Este discurso da patologização da transgeneridade reforça a resposta violenta social e se funda em bases biológicas profundas, relacionadas à presença de órgãos biológicos e “*decorre das concepções normativas pautadas no binarismo heterossexual que busca regular as sexualidades e subjetividades*” ^23^ (p. 8). Todo o processo transexualizador descrito em protocolos, portarias e guias médicos reforça, através dos procedimentos, a matriz heterossexual para os gêneros, possibilitando a regulamentação da vida pelo biopoder, regulando e modificando os processos de vida ^24^.

As trajetórias relatadas no curso do itinerário deste processo de modificação indicam que a clínica não é apenas um espaço de cuidado, mas um dispositivo biopolítico que seleciona e qualifica vidas por critérios normativos de gênero. Nos termos de Bento ^25^, isso opera como necrobiopoder, ao autorizar ou bloquear itinerários de transição e reconhecimento, produzindo zonas de inexistência social ou de exposição à violência.

Do ponto de vista da saúde coletiva latino-americana, a medicalização do gênero integra tecnologias de gestão da população - da organização do *corpus* documental (laudos, critérios de elegibilidade) à produção de bioidentidades legitimáveis - que convertem diferenças em desigualdades sanitárias ^26^. Conectando com a literatura latino-americana sobre violência e governo dos corpos, a leitura de Valencia ^27^ permite ver como regimes de mercado e segurança modulam quais corporalidades trans se tornam aceitáveis (e financiáveis) e quais permanecem relegadas à informalidade terapêutica e ao risco.

Independentemente de sua vivência com o processo de patologização agregado à construção social de seu gênero, percebe-se que muitas das entrevistadas não compreendem, em sua juventude, o que é ser uma travesti ou uma mulher trans, ou se reconhecem inicialmente dessa forma. De outra senda, com o passar dos anos, há participantes que já insistem em se identificarem como uma espécie de terceiro sexo. Outras, nem como trans ou travesti: como uma mulher. E, por último, há aquelas que negam o termo mulher:

“*Antigamente, eu me reconhecia como não binária. Mas, toda vez que a gente vai, no que eu ia numa loja e ia pegar o masculino, eu não via aquela roupa vestida em mim, não via aquele sapato vestido em mim, não gostava da minha voz*” (Turquesa, 21 anos).

“*Na verdade, na época, eu nem sabia o que era trans, travesti, eu sabia que eu era... que eu tinha uma identidade feminina, né?*” (Rubi, 30 anos).

“*E aí, sim, eu tive consciência de quem eu era. Que eu não era gay, que eu não era viado, que eu não era nada naquilo. Aqueles rótulos todos caíram por terra. Então, o que eu me identifiquei que eu sou uma trans. Nem travesti era, era uma trans mesmo, né? Aí veio toda essa mudança. Veio a minha autoafirmação na sociedade, na... comecei a me impor diante da minha família e tudo*” (Ametista, 51 anos).

“*‘Ah, eu sou trans’. Não, meu amor, eu sou mulher. Eu não sou mulher na genética, mas eu sou uma mulher, e não me trata como trans*” (Esmeralda, 46 anos).

“*A minha identidade sexual sempre foi tida. Desde pequena, eu sempre soube que gostava de homem. Mas, dizer que eu tinha uma mulher presa dentro de mim? Isso é ilusão da mente dessas pessoas, porque comigo não aconteceu desse jeito.* (...) *Eu sou um transexual, feminino, e nunca mulher*” (Jade, 46 anos).

Entendemos, a partir das contribuições de Jelín ^19^ e das articulações com a memória coletiva, que o processo pelo qual as pessoas “*se convertem*” em homens e mulheres é mediado pela aprendizagem das representações culturais de gênero, que são transmitidas, reiteradas, recordadas, organizadas a partir dos marcos sociais da memória (linguagem, espaços sociais, tempo, família, traduções e classes sociais). Portanto, a percepção sobre o que “*se é*” é construída de forma pessoal de acordo com atravessamentos individuais.

É interessante, entretanto, perceber que as falas sobre identidade transgênero, apesar de únicas, estão todas atreladas a uma pressuposição de senso comum do binômio feminino/masculino. Esta visão estereotipada, de acordo com Donna Haraway ^28^, faz parte de um sistema de classificação no formato de binômios: natureza/cultura, natureza/história, natural/humano, recurso/produto. Esta visão binária do sexo homem/mulher sustenta a construção biológica dos papéis sociais respaldadas em uma matriz heterossexual e reprodutiva, na qual a mulher sempre tem o papel voltado à reprodução, e sua personalidade é sempre relacionada à docilidade, à passividade e ao cuidado.

Portanto, a construção da identificação de gênero destas participantes, desde a sua juventude, perpassa também pela construção de um corpo alinhado com as expectativas de gênero, além da moldagem comportamental relacionado aos padrões hegemônicos das feminilidades, conforme será discutido no próximo item.

### Transformação comportamental e corporal

A identidade transgênero é entendida como um processo de construção atravessado, ao longo da vida, por estereótipos presentes em símbolos, como roupas, corpos, brincadeiras; além dos signos do que é ser mulher ou do que é ser homem, inclusive na própria linguagem usada pelas entrevistadas. A construção individual contínua da mulher transgênero perpassa por crenças e valores fortemente atrelados à ideia da mulher fruto de uma sociedade patriarcal. Suas aspirações, desejos e formas de apresentar-se geralmente transformam-se, ao longo do tempo, ao redor de características impostas pelo patriarcado às subjetividades femininas, como o imperativo da beleza, a predisposição ao amor, ao cuidado alheio, à delicadeza e à ternura, além relação direta entre ser mulher e funções biológicas e reprodutivas, conforme podemos ver nas falas a seguir:

“*Porque, assim, o que eu vejo muito nas trans, que eu acho errado... mas também isso é algo pessoal, comigo, né? Porque, o que é errado pra mim, pode não ser errado pro outro, né? Pode ser certo, mas é algo muito pessoal, é assim, eu falo: ‘gente, vocês não querem ser mulheres?’ Queremos. Mulher não se comporta desse jeito, mulher não fica falando alto no meio da rua, mulher não anda gesticulando o tempo todo, fazendo essas aberrações, gritando, tomando boca com homem. Mulher é ‘lady’. Mulher... ela sabe ser mulher. Mulher sabe chamar atenção sem precisar chamar tanto. Mesmo vestida de burca*” (Esmeralda, 46 anos).

“*Também não abro minha boca para ser mulher, porque eu não sou mulher. Mulher se nasce, não se transforma. E eu me transformei. Mulher é você, que pode gerar uma vida, botar no mundo uma vida, entendeu? Eu jamais vou poder botar uma vida no mundo, porque Deus não me deu esse direito. Então, como é que eu vou ter a pretensão de abrir a boca para dizer que eu sou mulher? Não! Eu sou um transexual, feminino, e nunca mulher, entendeu?*” (Jade, 46 anos).

Esta visão binária do sexo homem/mulher sustenta a construção biológica dos papéis sociais respaldadas em uma matriz heterossexual e reprodutiva, na qual a mulher sempre tem o papel voltado à reprodução, e sua personalidade é sempre relacionada à docilidade, à passividade e ao cuidado ^27^. As mulheres transgênero, embora transgressoras dessas normas imperativas de gênero, ao passo que rompem com esse sistema determinista, são coagidas a se submeter a essas ordens da vivência do que é ser mulher, pois necessitam assujeitar-se a uma estratégia de sobrevivência na sociedade. Ou seja, não há um rompimento de um modelo que se supõe a essas identidades e nem deveria ser imperativo essa demanda de transgressão, em vista do poder coercitivo das memórias coletivas entrelaçadas na sociedade ^19^. As identificações são processuais e contextuais; a memória registra arranjos práticos que equilibram proteção, pertencimento e afirmação.

A construção da identidade travesti e trans também tem como um dos pilares a transformação corporal, mediante incorporação de códigos de feminilidade (construção dos seios e quadril, eliminação dos pelos, modificação da voz), que podem, inclusive, ser mediados pelas tecnologias de modificações corporais (procedimentos cirúrgicos, uso de silicone industrial, uso de hormônios etc.). A partir dessas modificações, vai se construindo a (trans)identidade. O uso de hormônios, explicitado pelas entrevistadas, muitas vezes é introduzido precocemente:

“*Aí, com 15 anos, eu comecei a tomar hormônio, fiquei louca, tive vários problemas de saúde, horrível*” (Turquesa, 21 anos).

“*Ah, eu cheguei a tomar muito hormônio. O primeiro hormônio que eu tomei, eu tinha nove anos de idade*” (Água Marinha, 37 anos).

O fato de a maioria das entrevistadas iniciar o processo de hormonização muito cedo corrobora o estudo de Silva et al. ^29^, que, ao investigar sobre o processo de hormonização de mulheres trans e travestis, concluíram que 59,6% da amostra iniciou o uso de hormônios com menos de 18 anos de idade. Resultados similares foram encontrados na pesquisa de Krüger et al. ^30^, no qual observou-se que o início do uso dos hormônios se dava na média de 18,7 anos de idade na população de mulheres trans e travestis estudadas.

Sobre a questão da forma e da orientação de uso dos hormônios, todas as participantes relataram realizar automedicação ou receber orientação de seus pares. As travestis mais velhas, também apelidadas de “*mães de rua*”, são as que indicam o uso de hormônios e que também fazem a aplicação do silicone industrial. Além disso, são as responsáveis por transmitir a cultura e a memória coletiva/social desse grupo através da oralidade ^31^:

“*Quando a gente queria, é, começar, começava a se envolver com as travestis* (...) *nessas idas, eu comecei a ter contato, a conhecer, né, algumas, e algumas falavam: bixa, mas você é uma menininha. Você só precisa tomar hormônio. Ai, mas, onde que eu acho hormônio? Como é que é isso? Ai, eu não sabia nem como comprar. E um dia uma me deu uma cartela: você vai tomar. Você vai virar uma mocinha*” (Ametista, 51 anos).

Além disso, é possível inferir que o feminino expresso pelas mulheres trans ao longo de sua vida é constantemente negociado, reconstruído e agrega novos significados. Mas a máxima que acompanha esta constituição da identidade de gênero é o ideal de beleza feminina que toma contornos regionalizados, no qual mulheres trans e travestis brasileiras buscam construir um corpo quadris largos, glúteos fartos e seios grandes, sendo o uso de hormônios e demais tecnologias uma forma de fabricar uma beleza padrão.

Todas essas concepções do que seria a aparência feminina ou uma “*mulher de verdade*” são transmitidas a partir de uma memória coletiva, não apenas entre o grupo das transexuais e travestis, mas também nas outras correntes de memórias coletivas que se cruzam com as suas recordações, organizada a partir dos marcos sociais que reforçam modelos padrões das vivências do gênero e como esse gênero tem que estar impresso nos corpos.

Ademais, o processo que marca literalmente a transição e a assunção da identidade transgênero é o início das modificações corporais, explicitado nas falas de Água Marinha e Jade.

“*Quando eu botei meu peito, eu virei travesti*” (Água Marinha, 37 anos).

“*Nem sonhava em ser travesti na vida! Nem sonhava. Era um gay normal, como tem vários amigos aqui na cidade. Quem me conheceu na minha época de gay aqui na cidade, devido à necessidade, à fome, eu passei a me prostituir no Rio de Janeiro. Comecei a usar peruca na Avenida Atlântica, nunca quis saber de silicone; nada disso. Queria só me montar de noite para eu ganhar dinheiro para viver. Quando veio a oportunidade de ir embora para a Itália, pronto! Aí fui para a Itália, e, da Itália, foi que veio: botar um peito, matar a barba, fazer logo tudo. Depois de um tempo, eu sendo travesti, eu já não me aceitava como travesti, eu tirava o peito, deixava a barba crescer e voltava a ser gay, ou então mudava de sexo. Aí eu optei por mudar de sexo*” (Jade, 46 anos).

No que se refere à “*mudança de sexo*”, destacada como ponto final para Jade, observou-se sua relação com o termo “*trans*” entre as entrevistadas. Isso reflete contornos biomédicos, que tentam separar a travesti da mulher trans apontando que a travesti não deseja realizar a adequação à norma de gênero, ou seja, as modificações corporais, pois está sempre associada à violência e à marginalização. Isso se daria ao contrário da mulher trans que, a partir do diagnóstico, decide se adequar às normativas de gênero.

“*Antigamente, quando eu cheguei aqui em Conquista* [Vitória da Conquista]*, cheguei em 2001, voltei para cá esse ano, e eu era conhecida como Jade buceta. Porque, foi logo que eu tinha feito a cirurgia e o pessoal falava: ‘Olha, é travesti!’. E eu falava: ‘Travesti, não! Eu tenho buceta’*” (Jade, 46 anos).

As tecnologias corporais devem ser lidas como mediações: habilitam reconhecimento e agência, mas sob regras que delimitam o que conta como “*corpo legítimo*”. Esse duplo movimento ajuda a entender por que ruptura e acomodação coabitam as trajetórias.

Nesse contexto, percebe-se a formação de um ciclo na construção da identidade entre mulheres transgênero, que retomam, na performance de modelos pré-concebidos de comportamento e nomenclatura, as dúvidas e questionamentos iniciados e julgados na infância. Não obstante, o que percebemos é que as categorizações, na vivência real das travestis e transexuais, se entrecruzam e são difusas, não sendo possível estabelecer diferenciação. Assim, a indivídua escolhe como deseja nomear a sua identidade de acordo com a trajetória da construção de si, as memórias e marcos que conformam as suas recordações e a própria identidade.

Em suma, conclui-se que a experiência da transgeneridade dentro sistema sexo-gênero é dialético. Embora estas indivíduas borrem as fronteiras do sexo/gênero e rompam a visão hegemônica determinista e biológica, há um assujeitamento às normas de gênero e sexualidade impostas na sociedade, assumindo em seus atos sociais as perspectivas de gênero como uma tática de sobrevivência e pertencimento ao meio, o que é imputado a todas as pessoas, independente da sua identificação de gênero. Entretanto, entende-se que essas práticas se dão, de forma mais explícita e impositiva, para as pessoas que são entendidas dentro da sociedade como dissidências sexuais e de gênero, em uma tentativa mais pungente das tecnologias de gênero de controlar as subjetividades.

## Conclusão

Mostramos que as identificações de gênero na juventude trans são narradas em diálogo constante com quadros sociais e tecnologias corporais, sempre atravessadas pela regulação cis-heteronormativa. A contribuição central deste estudo é mobilizar Halbwachs para evidenciar como e por que certos passados se tornam dizíveis, revelando padrões de busca por legitimidade - seja por diagnósticos, seja pela adesão a padrões de feminilidade - sem reduzir a agência dessas experiências à ideia de mera transgressão. No contexto brasileiro, os achados indicam a urgência de despatologizar fluxos de cuidado, de reconhecer que cada vivência da transgeneridade é única e que não cabe em definições, e de alinhar políticas públicas aos itinerários concretos dessas trajetórias, que incluem hormonização precoce, redes de pares e barreiras territoriais. Destacamos como limitações do estudo o recorte territorial (contexto baiano) e temporal do trabalho de campo, adequados aos objetivos e explicitados para orientar leituras e usos dos resultados. Pesquisas futuras devem contrastar contextos de capitais e interiores em diferentes regiões do país e avaliar intervenções voltadas à redução de danos na hormonização e ao acesso informado ao cuidado.

## Data Availability

Os dados de pesquisa não estão disponíveis.
